# Differences Between Atypical and Typical Subtrochanteric Fractures Due to Low-Energy Trauma

**DOI:** 10.7759/cureus.90147

**Published:** 2025-08-15

**Authors:** Shuhei Hiyama, Hiroo Nakajima, Jiro Ando, Yoshiya Nibe, Hironao Shioiri, Yasuhiro Higai, Kenta Yanagisawa, Tomohiro Matsumura, Katsushi Takeshita

**Affiliations:** 1 Department of Orthopaedics, Jichi Medical University, Shimotsuke, JPN; 2 Department of Disaster Medicine, Jichi Medical University, Shimotsuke, JPN

**Keywords:** antiresorptive, fracture healing factors, intramedullary nails, s: osteoporosis, subtrochanteric fractures

## Abstract

Background: Subtrochanteric femur fractures caused by low-energy trauma are increasingly prevalent in aging populations. These fractures are categorized as atypical subtrochanteric fractures (ASF), often linked to antiresorptive therapy, or typical subtrochanteric fractures (TSF). This study aimed to compare the characteristics, surgical approaches, and clinical outcomes of ASF and TSF.

Methods: We conducted a retrospective cohort study at three hospitals in Japan, including 49 adult patients treated with cephalomedullary nails for low-energy subtrochanteric femur fractures between February 2007 and April 2024. Fractures were classified as ASF or TSF based on the American Society for Bone and Mineral Research (ASBMR) criteria. Patient demographics, surgical methods, and outcomes such as bone healing time and nonunion rate were evaluated and statistically compared.

Results: Among the 49 patients, 23 had ASF and 26 had TSF. The ASF group was significantly younger (69.0 ± 9.8 vs. 84.0 ± 7.3 years, p < 0.01) and had a higher prevalence of antiresorptive therapy (91.3% vs. 11.5%, p < 0.01). ASF patients more frequently underwent auxiliary plating (34.8% vs. 0%, p < 0.01) and received postoperative teriparatide. Despite these interventions, the ASF group exhibited significantly delayed healing (9.0 ± 1.9 vs. 5.8 ± 2.3 months, p < 0.01) and a higher nonunion rate (39.1% vs. 0%, p < 0.01).

Conclusion: ASF showed inferior healing outcomes compared to TSF, even with more aggressive treatments. These findings suggest that traditional TSF strategies may be inadequate for ASF, and biological augmentation, such as bone grafting, might be considered in initial management.

## Introduction

Subtrochanteric fractures account for approximately 5-20% of all proximal femoral fractures [1,2]. Among these, nearly two-thirds occur in individuals aged 50 years and older, who typically have compromised bone quality and sustain these fractures following low-energy trauma such as a simple fall [3,4]. As global populations continue to age, the incidence of low-energy subtrochanteric fractures is expected to rise accordingly [5]. Compared to trochanteric fractures, which have a reported revision rate of 3.3%, subtrochanteric fractures are associated with a higher revision rate ranging from 4.7% to 6.8% after intramedullary nail fixation [6,7]. This elevated risk may be due to several factors, including strong muscle forces acting on the fracture site, a higher likelihood of malalignment during surgery, impaired bone healing related to bisphosphonate use, and the relatively poor vascularity of the dense cortical bone in the subtrochanteric region [8,9]. To reduce postoperative complications, it is therefore crucial to obtain accurate fracture reduction during surgery and to use long intramedullary nails for stabilization [8-10].

Low-energy subtrochanteric fractures can be further classified into two distinct groups: typical subtrochanteric fractures (TSF) and atypical subtrochanteric fractures (ASF), the latter often occurring in patients undergoing long-term antiresorptive therapy [11]. Several retrospective reports have highlighted a higher frequency of complications and revision surgeries in patients with ASF following operative treatment [12]. Nonetheless, the specific differences in baseline characteristics and surgical outcomes between ASF and TSF remain insufficiently explored. This study aims to clarify these differences by directly comparing patient profiles and postoperative results between the two groups.

## Materials and methods

Study design, setting, and participants

This retrospective cohort study was carried out in the Department of Orthopaedic Surgery across three institutions (names withheld for peer review). Eligible participants included all patients treated for subtrochanteric femur fractures at these centers between February 2007 and April 2024. We conducted a thorough review of medical records and radiographs to collect clinical information and classify fracture types according to the Seinsheimer classification. This study was conducted in accordance with the ethical principles of the Declaration of Helsinki. Ethical approval was obtained from the institutional ethics committee (Approval ID: 20-158). The requirement for written informed consent was waived due to the retrospective nature of the study. However, information about the study was disclosed to the public on the hospital’s website, and patients were given the opportunity to opt out.

**Figure 1:**
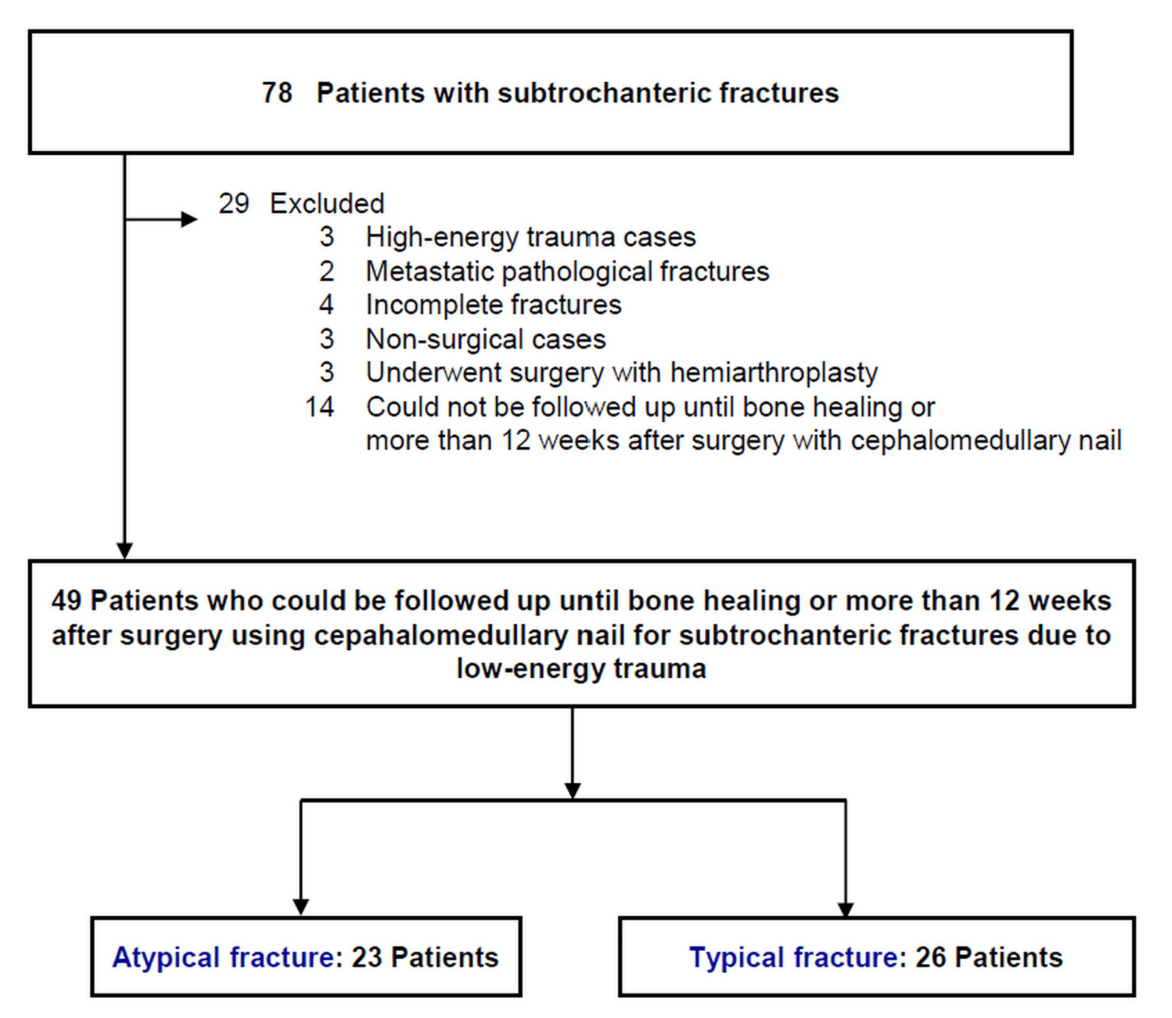
Patient selection A total of 78 patients with subtrochanteric femur fractures were initially identified. After applying the exclusion criteria—high-energy trauma (n = 3), metastatic pathological fractures (n = 2), incomplete fractures (n = 4), non-surgical cases (n = 3), surgeries with hemiarthroplasty (n = 3), and insufficient follow-up (n = 14)—49 patients who underwent cephalomedullary nailing for low-energy trauma and were followed up until bone healing or for at least 12 weeks postoperatively were included in the final analysis. These consisted of 23 patients with atypical subtrochanteric fractures (ASF) and 26 with typical subtrochanteric fractures (TSF).

Patients were excluded if they had sustained high-energy trauma, presented with metastatic pathological fractures or incomplete fractures, received non-surgical treatment, underwent surgical procedures without cephalomedullary nailing, or lacked adequate follow-up until radiographic union or at least 12 weeks postoperatively (Figure 1).

Only patients who sustained low-energy trauma (defined as a fall from standing height) and were managed with cephalomedullary nailing with sufficient follow-up were included.

All subtrochanteric fractures were screened using the 2010 ASBMR task force criteria at the time of study design and later re-evaluated based on the 2013 ASBMR definition to identify atypical femur fractures (AFF) [13,14]. Fractures meeting four or more of the five major diagnostic criteria were classified as ASF. In contrast, osteoporotic subtrochanteric fractures that did not meet these criteria were categorized as TSF.

Surgical procedure

All operations were performed under general or lumbar anesthesia with patients positioned supine on a traction table under fluoroscopic guidance. Fracture fixation was achieved using open or closed reduction and internal fixation. In cases where an additional plate was used [15], a monocortical locking plate was applied on the tension side after fracture alignment.

A skin incision was made proximal to the greater trochanter to allow insertion of the nail attached to the aiming device. After positioning the lag screw in the femoral neck, distal locking screws were inserted through the static holes. In all cases, the lag screw was locked. Decisions regarding implant type, nail length and diameter, number of distal locking screws, and use of additional fixation (e.g., auxiliary plates or cerclage wiring), as well as timing of postoperative weight-bearing, were left to the discretion of the operating surgeon.

Data collection and measurements

Preoperative data collected included age, sex, body mass index (BMI), American Society of Anesthesiologists (ASA) physical status classification, baseline ambulatory status, fracture classification (Seinsheimer), and history of antiresorptive therapy. Walking ability was assessed using a 5-point scale: 5 = independent walking; 4 = walking with a cane; 3 = ambulating between parallel bars; 2 = walker-dependent; and 1 = wheelchair-dependent [16]. Intraoperative and perioperative data included nail length, use of auxiliary plate and cerclage wiring, operative duration, intraoperative blood loss, blood transfusion volume, history of antiresorptive therapy, and use of teriparatide. Nails shorter than 240 mm were categorized as short, and those longer than 240 mm were classified as long.

Postoperative outcomes evaluated were the immediate neck-shaft angle (NSA), residual translational displacement at the fracture site, time to radiographic union, nonunion, peri-implant fractures, and reoperations. NSA was calculated as the angle between a line along the femoral neck axis and a line parallel to the anatomical axis of the femoral shaft. Residual displacement was measured using postoperative anteroposterior and lateral radiographs. Fracture union was defined as bridging callus across at least three out of four cortices on orthogonal radiographs [12,15,17]. Nonunion was diagnosed if union had not occurred within one year after surgery or if implant failure (e.g., nail breakage) was observed during follow-up.

Statistical analysis

A comparative analysis was conducted between the ASF and TSF groups. Continuous variables were compared using the Mann-Whitney U test, and categorical variables were compared using the chi-square test. A p-value of < 0.05 was considered statistically significant. Continuous and ordinal variables were dichotomized before statistical comparison. In the analysis of time to union, patients diagnosed with nonunion (n = 9) were excluded. Categorical data are presented as percentages, and continuous data are reported as means with standard deviations. All statistical analyses were performed using IBM Corp. Released 2017. IBM SPSS Statistics for Windows, Version 22. Armonk, NY: IBM Corp.

## Results

Among 78 patients with subtrochanteric fractures, 49 patients were included in the analysis, and there were 23 cases of ASF and 26 cases of TSF (Figure 1). Demographic characteristics and perioperative findings are summarized in Table 1. In patient characteristics, there were significant differences in average age (69 ± 9.8 years vs. 84 ± 7.3 years, p < 0.01) and a history of antiresorptive therapy (91.3% vs. 11.5%, p < 0.01). In perioperative measurements, there were significant differences in the use of cephalomedullary nailing with auxiliary plate (34.8% vs. 0%, p < 0.01), mean surgical time (141 ± 66 min vs. 114 ± 46 min, p = 0.04), and postoperative teriparatide treatment (56.5% vs. 11.5%, p = 0.01). In postoperative outcomes (Table 2), there were significant differences in residual displacement in the AP view (1.4 ± 2.1 mm vs. 3.0 ± 3.9 mm, p < 0.01), the mean bone union time (9.0 ± 1.9 months vs. 5.8 ± 2.3 months, p < 0.01), and the rate of nonunion (39.1% vs. 0%, p < 0.01).

**Table 1:** Patient characteristics and perioperative measurements Values are shown as numbers (%) for categorical variables and mean ± standard deviation (SD) for continuous variables. Continuous variables were analyzed using the Mann–Whitney U test (ᵃ), and categorical variables were analyzed using the chi-square test (ᵇ). Test statistics (U or χ² values) are reported in the "Test statistic" column. A p-value of < 0.05 was considered statistically significant and is indicated with * (p < 0.05) or ** (p < 0.01). ᵃ Mann–Whitney U test ᵇ Chi-square test ASF: atypical subtrochanteric fracture, TSF: typical subtrochanteric fracture, ASA: American Society of Anesthesiologists physical Status classification system

	ASF(n=23)	TSF(n=26)	Test statistic	p-value
Patient characteristics and mesurements	n (%)	n (%)		
Mean age (years) , mean ± SD	69 ± 9.8	84.3 ± 7.3	U = 170.4^a^	<0.01**
Sex			X^2 ^= 1.12^b^	0.29
Male	2 (8.7)	4 (15.4)		
Female	21 (91.3)	21(84.6)		
Body mass index (kg/m^2^), mean ± SD	24.3 ± 4.9	22.1 ± 4.1	U = 227.1^a^	0.15
ASA score (point)				
1	2(8.7)	0(0)	X^2 ^= 3.11^b^	0.08
2	19(82.6)	18(69.2)		
3	2(8.7)	8(30.8)		
Preoperative waking ability (point)			X^2 ^= 3.06^b^	0.08
5	20(87.0)	16(61.6)		
4	3(13.0)	5(19.2)		
3	0	5(19.2)		
2	0	0		
1	0	0		
Seinsheimer Classification			X^2 ^= 3.28^b^	0.07
1	0(0)	0(0)		
2	19(82.6)	14(53.8)		
3	4(17.4)	12(46.2)		
4	0(0)	0(0)		
5	0(0)	0(0)		
Nail length			X^2 ^= 0.00^b^	1
Short nail	6(26.0)	7(26.9)		
Long nail	17(74.0)	19(73.1)		
Implant			X^2 ^= 6.63^b^	0.01*
Cephalomeddullary nail	15(65.2)	26(100.0)		
Cephalomeddullary nail with auxiliary plate	8(34.8)	0(0)		
Wiring	3(13.0)	8(30.8)	X^2 ^= 3.71^b^	0.054
Antiresorptive therapy	21(91.3)	3(11.5)	X^2 ^= 7.12^b^	<0.01**
Surgical time (min), mean ± SD	141±66	114±46	U = 196.5^a^	0.04*
Operative blood loss(ml), mean ± SD	307±365	288±206	U = 255.2^a^	0.38
Amount of blood transfusion(U), mean ± SD	1.2±1.7	2.4±2.3	U = 270.6^a^	0.57
Teriparatide treatment	13(56.5)	3(11.5)	X^2 ^= 6.77^b^	0.01*

**Table 2:** Postoperative outcomes Comparison of postoperative outcomes between patients with atypical (ASF) and typical (TSF) subtrochanteric femur fractures. Values are presented as numbers (%) for categorical variables and mean ± standard deviation (SD) for continuous variables. Nine nonunion cases in the ASF group were excluded from the analysis of time to bone union. Continuous variables were analyzed using the Mann–Whitney U test (ᵃ), and categorical variables were analyzed using the chi-square test (ᵇ). Test statistics (U or χ² values) are reported in the "Test statistic" column. A p-value of < 0.05 was considered statistically significant and is indicated with * (p < 0.05) or ** (p < 0.01). ᵃ Mann–Whitney U test ᵇ Chi-square test ASF: atypical subtrochanteric fracture, TSF: typical subtrochanteric fracture

	ASF(n=23)	TSF(n=26)	Test statistic	p-value
Parameter	n (%)	n (%)		
Neck shaft angle (degree)	130.7±5.8	133.0±5.9	U = 241.6^a^	0.25
Residual displacement AP view(mm)	1.4±2.1	3.0±3.9	U = 170.4^a^	<0.01**
Residual displacement lateral view (mm)	3.4±3.6	3.2±4.7	U = 249.4^a^	0.32
Bone union time (months)*	9.0±1.9	5.8±2.3	U = 91.2^a^	<0.01**
Nonunion	9 (39.1)	0(0)	X^2 ^= 6.63^b^	<0.01**
Peri-implant fracture	1 (4.3)	3 (11.5)	X^2 ^= 0.36^b^	0.55
Reoperation	3(13.0)	1(3.9)	X^2 ^= 1.12^b^	0.29

## Discussion

The present study investigated the differences in patient characteristics and postoperative outcomes between the ASF group and the TSF group. This study revealed that the ASF group had longer bone union time and a higher incidence of nonunion, despite a younger mean age and the use of more augment plates and more teriparatide than the TSF group. These findings suggest that treatment of ASF is not established, and we must consider additional devices, such as the use of bone grafting for the fracture site.

Atypical femur fractures (AFF) differ fundamentally from typical subtrochanteric fractures in their underlying biology and mechanism. AFFs are strongly associated with long-term bisphosphonate use, which markedly suppresses bone turnover and alters bone quality [18]. In addition, a retrospective study showed that patients with AFF were the most frequent in the 65-84-year-old age group [19]. In our study, average age at injury was also lower in the ASF group than the TSF group, and most cases with ASF continued antiresorptive therapy. We should consider atypical fracture when diagnosing subtrochanteric fracture in younger patients with the use of antiresorptive medications. The suppression of bone remodeling in AFFs not only predisposes to fracture but also impairs the healing process by limiting osteoclastic resorption and new bone formation [18]. Consequently, AFFs tend to heal more slowly and unpredictably than typical fractures.

Clinical series have reported that bisphosphonate-related subtrochanteric fractures take on average about 12 months to unite, versus ~4-6 months in typical subtrochanteric fractures [20]. Therefore, treatment for AFF requires a strategy that accounts for delayed bone healing, including careful implant selection. Importantly, the implant must endure cyclical loads over a much longer period. Prolonged healing increases the risk of hardware fatigue or failure (e.g., nail or screw breakage) before union occurs, especially if the fixation construct is not sufficiently robust.

The first choice of implant for internal fixation of subtrochanteric fracture is the intramedullary nail, which requires less operating time and blood loss, has good functional results, and has a low revision rate [21]. The intramedullary nails are mechanically superior to extramedullary implants [22]. In ASF, the result of extramedullary implants is also poor, with a 29% implant failure rate and a 38% revision rate [23]. On the other hand, in a case series investigating postoperative outcomes after cephalomedullary nailing of 48 cases with ASF, the nonunion and delayed union rate was 31.3%, and the mean time to union was 10.7 months [17]. In the present study, the ASF group used more augment plates, which might lead to less residual displacement in the anterior-posterior (AP) view than the TSF group. Moreover, the ASF group used more teriparatide postoperatively than the TSF group. However, the ASF group had a longer bone union time and a higher incidence of nonunion than the TSF group. Moreover, the subtrochanteric location itself appears to confer a higher risk of nonunion among AFFs-a recent meta-analysis found a nonunion rate of approximately 15% for subtrochanteric AFFs versus 4% for mid-diaphyseal AFFs [24]. Based on these findings, it is possible that ASF may not keep a stable postoperative outcome with standard treatment of TSF alone. Indeed, Egol and colleagues have emphasized that achieving stable alignment and using durable fixation is critical, as even slight malreduction or residual gaps in AFF can prolong healing significantly [25].

In cases of extreme femoral bowing or other anatomic constraints (e.g., a narrow medullary canal or previous implants in situ), plate fixation can be considered as an alternative [18]. However, plating in AFF comes with its own limitations: a plate provides less optimal load-sharing (and more bending moment) than an intramedullary nail (IM) in this region, and a long plate may be required to distribute stress. Because AFF bone is often brittle with suppressed remodeling, stress concentration at the end of a plate can precipitate a new fracture or hardware failure [26-28]. Additionally, compression plating relies on direct (primary) bone healing, which is inefficient in AFF due to impaired osteoclastic activity [29]. Intramedullary nails confer biomechanical advantages in AFF by virtue of their medial location (closer to the weight-bearing axis) and load-sharing capacity, which permits controlled micro-motion and secondary bone healing (callus formation) [29]. However, the narrow medullary canal in ASF poses a significant challenge in selecting appropriate IM nails. Detailed morphological considerations, such as the diameter and neck-shaft angle of the intramedullary nail, are critical. ASF often involves a narrow medullary cavity, and the insertion of a standard-diameter nail may be technically difficult or biomechanically suboptimal. Therefore, surgeons must carefully select nail diameter and neck-shaft angle to ensure optimal fit and stability in ASF cases.

Oh et al. reported that the fracture surface of ASF was devoid of cells, suggesting an inhibited state of bone metabolic turnover and a high risk of prolonged healing due to a marked decrease in biological activity [30]. Therefore, the authors reported stable results with treatment by internal fixation with intramedullary nails and autologous cancellous bone graft harvested from the greater trochanter at the entry site of an intramedullary nail using a crown reamer [30]. In addition to optimal reduction quality for achieving mechanical stability and internal fixation by cephalomedullary nails, bone grafting for the fracture site might be needed for the initial surgery of ASF. Therefore, while bone grafting is not routinely needed for typical subtrochanteric fractures (which usually heal with fixation alone), it can be a valuable adjunct in atypical fractures to biologically stimulate union. Indeed, Yoon et al. note that factors like implant choice and bone graft use can substantially influence healing outcomes in AFF, contributing to the wide variability in reported union rates [24].

This study had several limitations. First, the sample size of subtrochanteric fractures was relatively small, which may be due to the low incidence of this fracture type. Second, osteoporosis was not evaluated by bone mineral density and bone metabolism markers. Third, the duration of antiresorptive therapy and its starting age were not investigated. Finally, decisions regarding the type of implant, the length and diameter of the nail, the number of distal locking screws, the use of augmentation devices such as plates or cerclage wiring, and the timing of postoperative weight-bearing were made at the discretion of the operating surgeon.

## Conclusions

We retrospectively analyzed differences in surgical outcomes between ASF and TSF. Despite being younger, ASF patients exhibited significantly longer healing times and higher nonunion rates. These findings suggest that standard treatments for TSF may be inadequate for ASF, and additional strategies, such as bone grafting, might be considered in initial management.
